# Quality of life management capability in elderly frail patients with colorectal cancer: a latent profile analysis

**DOI:** 10.3389/fonc.2026.1826323

**Published:** 2026-09-10

**Authors:** Wenyu Chai, Na Wang, Ti Wang, Yue Xiang, Gamei Li

**Affiliations:** 1 Gansu Maternal and Child Health Care Hospital (Gansu Provincial Central Hospital), Lanzhou, Gansu, China; 2 Gansu University of Chinese Medicine, Lanzhou, Gansu, China

**Keywords:** cancer nursing, colorectal cancer, frailty, potential profile analysis, quality of life

## Abstract

**Objective:**

To explore the potential categories and influencing factors of quality of life management capability in elderly frail patients with colorectal cancer using latent profile analysis.

**Methods:**

This cross-sectional study used convenience sampling to recruit patients from three tertiary hospitals between October 2023 and May 2025. Data were collected using multiple-scale surveys. Latent profile analysis (LPA) was used to identify potential categories and analyze influencing factors.

**Result:**

A total of 280 patients were included. Quality of life management capability was divided into three categories: the low quality of life, low emotional level group (20.35%); the medium quality of life, low social function group (39.28%); and the high quality of life, high role management group (40.35%). Multivariable logistic regression showed that educational level, tumor stage, enterostomy status, family income, sex, disease acceptance, emotional level, self-efficacy, and social support level were the main influencing factors (*P* < 0.05).

**Conclusion:**

There is significant heterogeneity in the quality of life management capability of patients. Personalized interventions should be implemented based on category characteristics to improve quality of life.

## 1 Introduction

Frailty is a complex, aging-related issue that renders patients more vulnerable to stressors and prone to adverse health outcomes (1). Studies indicate that the incidence of preoperative frailty in colorectal cancer (CRC) patients reaches 43.8%, with a postoperative complication risk approximately 50% higher than that of non-frail patients. The prevalence of frailty among elderly CRC patients ranges between 14% and 40%, and the mortality risk in frail patients is two to three times higher than that in non-frail patients (2). This condition leads to prolonged hospital stays, an increased risk of postoperative complications, and consequently impaired quality of life (QOL) (3). Self-management behavior plays a crucial role in the health management of cancer patients and serves as a key approach to improving physical condition and optimizing ultimate health outcomes (4). However, the majority of existing studies evaluate quality of life based on the total score of scales, ignoring inter-individual heterogeneity, and there is a lack of research focusing on elderly frail patients with colorectal cancer. Latent profile analysis (LPA) explains the relationships between external continuous variables through latent categorical variables, enabling the differentiation of manifest variables (5). This study employed LPA to explore the latent categories of quality of life management capabilities in this population and their influencing factors, aiming to provide a basis for formulating personalized quality of life management plans. Given the cross-sectional design of this study, the regression analyses presented are exploratory in nature and are intended only for hypothesis generation; causal inferences cannot be drawn from the results.

## 2 Subjects and methods

### 2.1 Study subjects

This was a cross-sectional study. Convenience sampling was used to select elderly frail patients with colorectal cancer from three tertiary hospitals between October 2023 and May 2025. This study received ethical approval from the hospital (Approval No.: GSFY Ethics Review (21)).

#### 2.1.1 Inclusion criteria

① Aged ≥ 60 years;

② Pathologically confirmed Tis-III stage colorectal cancer;

③ Fried frailty phenotype score ≥ 1;

④ Completed primary treatment and entered the follow-up period.

#### 2.1.2 Exclusion criteria

① Cognitive impairment or mental illness;

② History of other malignant tumors;

③ Severe complications or comorbidities.

### 2.2 Survey instruments

#### 2.2.1 General information questionnaire

A self-designed general information questionnaire was used to collect data on sex, marital status, place of residence, living arrangement, educational level, average monthly household income, and other demographic characteristics.

#### 2.2.2 European organization for research and treatment of cancer quality of life questionnaire–core 30

Developed by the European Organization for Research and Treatment of Cancer (6), this questionnaire, which is used to assess the quality of life of cancer patients, consists of 30 items covering five functional domains, nine symptom domains, and one overall health status domain. The item scores are converted into standardized scores ranging from 0 to 100. Higher scores in the functional and overall health domains indicate better quality of life, while higher scores in the symptom domains indicate poorer quality of life. If the completion rate for a scale with missing items was ≥50%, the score was calculated based on the average of the completed items. The Cronbach’s α coefficient for this scale in the present study was 0.959.

#### 2.2.3 Strategies used by people to promote health (SUPPH) scale

Developed by Lev et al. (7), this scale is used to measure the confidence level of cancer patients in managing their disease. In this study, the Chinese version of the scale, translated and revised by Qian Huijuan et al. (8), was adopted. The scale includes three dimensions (positive attitude, self-stress reduction, and self-decision-making) and 28 items. Each item is scored using a 5-point Likert scale, with a total score ranging from 28 to 140. A higher score indicates stronger self-efficacy. The Cronbach’s α coefficient for this scale in the present study was 0.976.

#### 2.2.4 Chinese version of the acceptance illness scale (AIS-CHI)

Originally developed by Felton et al. (9), the Chinese version of the scale, translated and revised by Zhao Wenwen (10), was used in this study. It consists of eight items, each scored using a 5-point Likert scale, with a total score ranging from 8 to 40. A score <20 indicates a low level of illness acceptance, a score of 20–30 indicates a moderate level, and a score >30 indicates a high level. The Cronbach’s α coefficient for this scale in the present study was 0.893.

#### 2.2.5 Hospital anxiety and depression scale

Developed by Zigmond et al. (11), the Chinese version of the scale, translated and revised by Ye Weifei et al. (12), was adopted in this study. It includes two subscales (anxiety and depression) with a total of 14 items. Each item is scored using a 4-point Likert scale, and the total score of each subscale ranges from 0 to 21. A score >7 indicates the presence of anxiety or depression in the patient. The overall Cronbach’s α coefficient for this scale in the present study was 0.932.

#### 2.2.6 Social support rating scale

Compiled by Xiao Shuiyuan (13), this scale is used to measure the level of social support received by an individual. It includes three dimensions (objective support, subjective support, and social support utilization) and 10 items. The total score ranges from 12 to 66, and a higher score indicates a higher level of social support received by the individual. The Cronbach’s α coefficient for this scale in the present study was 0.925.

### 2.3 Data collection and quality control methods

The survey was conducted one week after the patients were discharged from the hospital upon completion of their primary treatment plan. Two uniformly trained researchers distributed the questionnaires online. Before the survey, the researchers used a standardized guideline to obtain informed consent from the patients and their family members. After the questionnaires were collected, missing and incorrect entries were checked in a timely manner to ensure the quality of the filled information.

### 2.4 Statistical methods

Mplus 8.3 software was used for LPA. Starting from the assumption of one category, the number of categories was gradually increased. The Akaike Information Criterion (AIC), Bayesian Information Criterion (BIC), and adjusted Bayesian Information Criterion (aBIC) were used to evaluate the model fit, with lower values indicating a better fit. Entropy was used to measure the classification accuracy, with values closer to 1 indicating more accurate classification. The Lo-Mendell-Rubin adjusted likelihood ratio test (LMRT) and Bootstrap likelihood ratio test (BLRT) were used to compare the fit differences between latent category models. *P* < 0.05 indicated that the model with k categories was more optimal, and this was used to select the best model.

SPSS 26.0 software was used for data analysis. Categorical variables were described using frequencies and percentages, while continuous variables were expressed as mean ± standard deviation (SD). Based on the best model, chi-square tests and one-way analysis of variance (ANOVA) were used to compare differences in general information, self-efficacy, and other aspects among patients in different latent categories. Binary logistic regression was used for further analysis, with *P* < 0.05 considered statistically significant.

For multivariable logistic regression analysis, the following procedures were performed. First, collinearity diagnostics were conducted using the variance inflation factor (VIF); all continuous variables had VIF <2, indicating no significant multicollinearity. Second, univariable analyses were performed for all candidate variables, and variables with *P* < 0.1 were considered for inclusion in the multivariable model. Third, a stepwise backward elimination procedure based on AIC was applied to select the final set of predictors. A two-tailed *P* < 0.05 was considered statistically significant in the final multivariable model. As this was a cross-sectional study, regression analyses were used only for hypothesis generation and exploratory identification of potential factors; causal inferences cannot be drawn. Categorical variables were assessed using cross-tabulation and generalized VIFs, and no severe multicollinearity was detected.

## 3 Results

### 3.1 General characteristics of the study participants

Based on the principle that the sample size for regression analysis should be at least 15 times the number of independent variables, and considering for a 10% missing-data rate, 17 independent variables were included, resulting in a required sample size of 280 participants. A total of 291 questionnaires were distributed, and 280 valid questionnaires were returned, yielding an effective response rate of 96.21%. The low QOL group had a higher proportion of women (68.42%) and rural residents (82.45%) than the high QOL group (46.91% and 34.51%, respectively). Patients with primary school education or below were overrepresented in the low QOL group (64.91%) and underrepresented in the high QOL group (27.43%). The general characteristics of the participants are presented in **Table 1**.

**Table 1:** Univariate analysis of general characteristics and quality of life management profiles.

Characteristic	Total number (n = 280)	Low QOL, low emotional level group (20.35%)	Moderate QOL, low social function group (39.28%)	High QOL, high role management group (40.35%)	*P value*
Sex, n (%)					0.029
Male	129 (46.07)	18 (31.57)	51 (46.36)	60 (53.09)	
Female	151 (53.92)	39(68.42)	59(53.63)	53(46.91)	
Marital status, n (%)					0.428
Married	253 (90.35)	53 (92.98)	101 (91.81)	99 (87.61)	
Others	27 (9.65)	4 (7.01)	9 (8.18)	14 (12.38)	
Place of residence, n (%)					0.001
Rural	171 (61.07)	47 (82.45)	85 (77.27)	39 (34.51)	
Urban	109 (38.92)	10 (17.54)	25 (22.72)	74 (65.48)	
Living arrangement, n (%)					0.027
Living alone	13 (4.64)	3 (5.26)	6 (5.45)	4 (3.53)	
Living with spouse only	128 (45.71)	16 (28.07)	59 (53.63)	53 (46.90)	
Living with children	139 (49.64)	38 (66.66)	45 (40.90)	56 (49.55)	
Educational level, n (%)					0.001
Primary school and below	117 (41.78)	37 (64.91)	49 (44.54)	31 (27.43)	
Junior high school	77 (27.50)	16 (28.07)	28 (25.45)	33 (29.20)	
Senior high school	67 (23.92)	4 (7.01)	27 (24.54)	36 (31.85)	
College and above	19 (6.78)	0 (0.00)	6 (5.45)	13 (11.50)	
Per capita monthly family income, n (%)					0.001
< 3000 RMB	79 (28.21)	18 (31.57)	29 (26.36)	32 (28.31)	
3000–6000 RMB	131 (46.78)	30 (52.63)	64 (58.18)	37 (32.74)	
> 6000 RMB	70 (25.00)	9 (15.78)	17 (15.45)	44 (38.93)	
Payment method, n (%)					0.097
Urban-rural resident medical insurance	154 (55.00)	34 (59.64)	63 (57.27)	57 (50.44)	
Employee medical insurance/commercial insurance	97 (34.64)	13 (22.80)	38 (34.54)	46 (40.70)	
Self-payment	29 (10.35)	10 (17.54)	9 (8.18)	10 (8.84)	
Tumor Stage, n (%)					0.001
Tis stage	72 (25.71)	8 (14.03)	18 (16.36)	46 (40.70)	
Stage I	83 (29.64)	11 (19.29)	32 (29.09)	40 (35.39)	
Stage II	67 (23.92)	24 (42.10)	38 (34.54)	5 (4.42)	
Stage III	58 (20.71)	14 (24.56)	22 (20.00)	22 (19.46)	
Intestinal stoma, n (%)					0.001
Yes	132 (47.14)	47 (82.45)	67 (60.90)	18 (15.9)	
No	148 (52.85)	10 (17.54)	43 (39.09)	95 (84.07)	
Disease duration, n (%)					0.132
≤.1 months	122 (43.57)	23 (40.35)	56 (50.91)	43 (38.05)	
>12 months	158 (56.42)	34 (59.64)	54 (49.09)	70 (61.94)	
Radiotherapy, n (%)					0.065
Yes	76 (27.14)	14 (24.56)	23 (20.90)	39 (34.51)	
No	204 (72.85)	43 (75.43)	87 (79.09)	74 (65.48)	
SUPPH score	102.19 ± 21.28	76.10 ± 24.62	107.29 ± 21.38	110.77 ± 14.21	0.006
SSRS score	41.19 ± 0.47	37.96 ± 8.20	41.44 ± 6.19	42.58 ± 3.24	0.009
HADS score	6.47 ± 1.67	9.94 ± 2.35	4.23 ± 3.26	4.83 ± 1.20	0.001
AIS-CHI score	26.15 ± 5.27	17.42 ± 4.40	24.22 ± 3.32	33.20 ± 3.35	0.008

### 3.2 Results of latent profile analysis of quality of life management capability

LPA was conducted to identify latent profiles of quality of life management capability among elderly frail patients with colorectal cancer. Models containing one to four latent profiles were evaluated sequentially (**Table 2**). The results showed that Model 3 had the lowest Akaike Information Criterion (AIC), Bayesian Information Criterion (BIC), and adjusted Bayesian Information Criterion (aBIC) values, with an entropy value of 0.998. Additionally, both the Lo-Mendell-Rubin adjusted likelihood ratio test (LMRT) and the Bootstrap likelihood ratio test (BLRT) reached statistical significance (*P* < 0.05). Therefore, Model 3 was identified as the optimal model.

**Table 2:** Fit indices of latent profile models for quality of life management capability in elderly frail patients with colorectal cancer (n = 280).

Number of profiles	AIC	BIC	aBIC	Entropy	LMR	BLRT	Profile probability
1	10752.543	10823.775	10772.977	−	−	−	−
2	6891.786	7003.087	6923.715	0.996	0	0.001	0.31812/0.68188
3	6501.426	6732.933	6567.838	0.998	0.0316	0.001	0.10324/0.2151/0.68167
4	6808.97	6960.339	6852.392	0.943	0.0105	0.001	0.22612/0.09188/0.45531/0.22669

**Figure 1:**
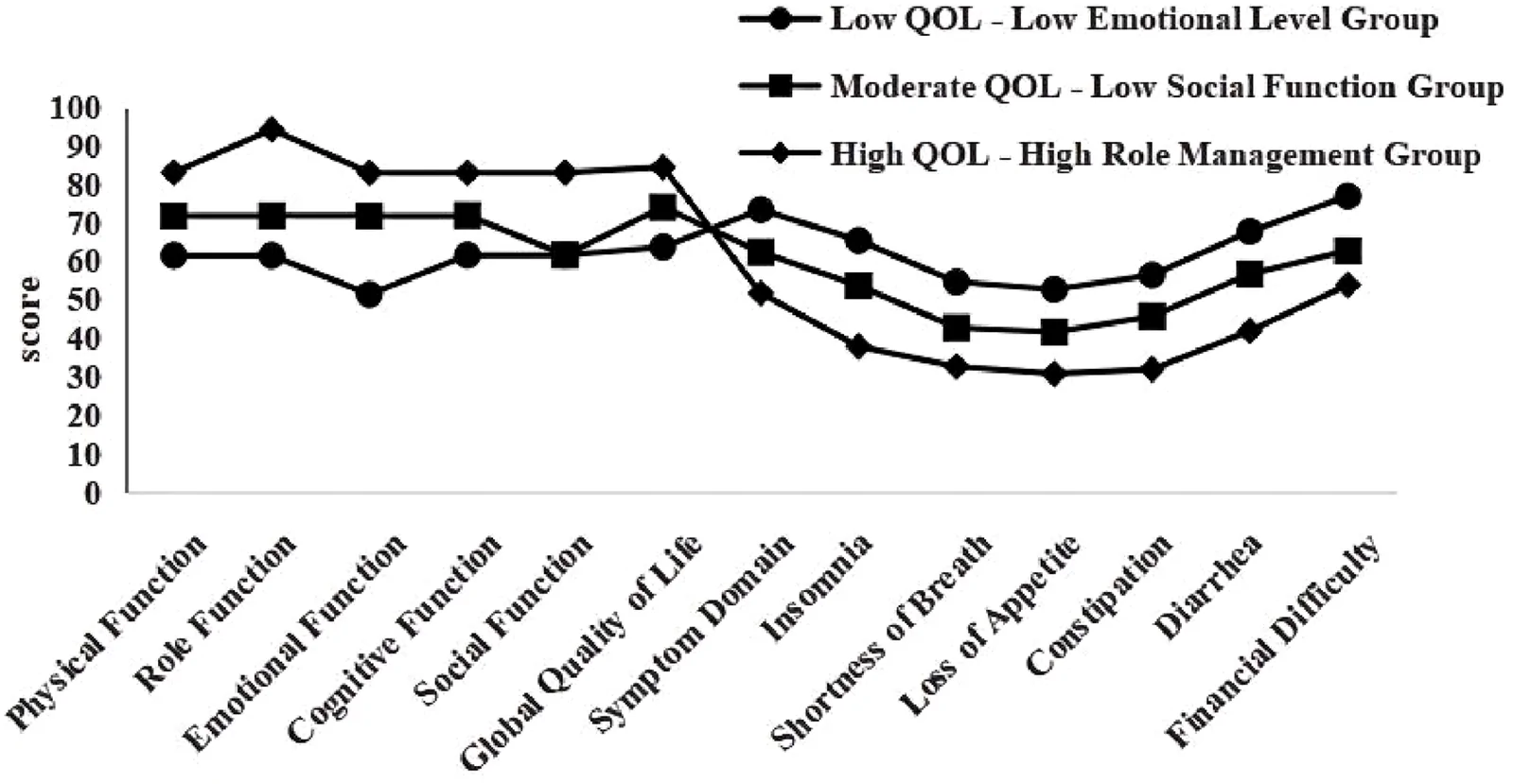
Characteristic distribution map of potential categories of quality of life management capability in elderly frail patients with colorectal cancer..

Based on the mean scores of the 15 dimensions in the European Organization for Research and Treatment of Cancer Quality of Life Questionnaire-Core 30 (EORTC QLQ-C30), the latent profiles of quality of life management capability in elderly frail patients with colorectal cancer were derived (**Figure 1**). Each profile was named according to the manifest characteristics of the scale dimensions:

Profile 1 (n = 57, 20.35%): The overall quality of life management capability of this group was lower than that of the other two groups, with the lowest score in the emotional dimension. Thus, this profile was designated the “low QOL, low emotional level group”.

Profile 2 (n = 110, 39.28%): The self-management capability of this group was moderate, but the score in the social function dimension was low. It was therefore designated the “moderate QOL, low social function group”.

Profile 3 (n = 113, 40.35%): The self-management capability of this group was higher than that of the other two groups, with the highest score observed in the role function dimension. It was designated the “high QOL, high role management group”.

### 3.3 Quality of Life management capability scores across different latent profiles

Statistically significant differences were observed in the total scores and item scores of quality of life management capability among the three latent profiles of elderly frail patients with colorectal cancer (*P* < 0.05). Notably, the high QOL group scored highest in role function (93.3 ± 2.5), while the low QOL group had the lowest score in emotional function (51.1 ± 10.2). The symptom domain scores were highest in the low QOL group (73.7 ± 12.4) and lowest in the high QOL group (53.2 ± 7.3). Detailed results are presented in **Table 3**.

**Table 3:** Quality of life management behavior scores across different latent profiles in elderly frail patients with colorectal cancer (mean ± SD).

Domain	Low QOL, low emotional level group (20.35%)	Moderate QOL, low social function group (39.28%)	High QOL, high role management group (40.35%)	F	*P*
Physical function	61.8 ± 12.3	71.8 ± 10.6	83.1 ± 8.4	70.338	<0.001
Role function	61.6 ± 11.3	71.84 ± 13.6	93.3 ± 2.5	59.646	<0.001
Emotional function	51.1 ± 10.2	71.90 ± 14.8	83.0 ± 6.4	51.456	<0.001
Cognitive function	61.4 ± 12.5	71.73 ± 8.5	83.2 ± 9.4	29.500	<0.001
Social function	61.1 ± 13.8	61.64 ± 0.5	83.9 ± 4.9	43.142	<0.001
Symptom domain	73.7 ± 12.4	61.2 ± 10.3	53.2 ± 7.3	80.581	<0.001
Global quality of life	64.5 ± 12.1	73.7 ± 13.2	84.7 ± 10.3	23.766	<0.001
Insomnia	65.5 ± 8.7	53.8 ± 10.2	37.3 ± 11.3	46.824	<0.001
Shortness of breath	54.4 ± 8.3	42.89 ± 10.3	32.6 ± 9.6	16.775	<0.001
Loss of appetite	52.6 ± 10.2	45.5 ± 14.6	30.4 ± 12.3	96.876	<0.001
Constipation	56.3 ± 9.3	65.4 ± 14.4	31.8 ± 7.5	45.956	<0.001
Diarrhea	68.2 ± 3.4	26.4 ± 12.4	81.9 ± 10.6	75.856	<0.001
Financial difficulties	76.1 ± 14.4	62.7 ± 8.4	56.3 ± 11.5	65.886	<0.001

### 3.4 Univariate analysis of latent profiles of quality of life management capability

Significant differences among the three latent profiles were observed with respect to sex, place of residence, living arrangement, educational level, per capita monthly family income, tumor stage, enterostomy status, SUPPH score, SSRS score, HADS score, and AIS-CHI score (*P* < 0.05; **Table 1**). For the binary logistic regression analyses, univariable analyses showed that variables with *P* < 0.1 included sex, place of residence, living arrangement, educational level, family income, tumor stage, enterostomy status, SUPPH score, SSRS score, HADS score, AIS-CHI score, payment method (*P* = 0.097), and radiotherapy status (*P* = 0.065).

### 3.5 Multivariable analysis of latent profiles of quality of life management capability

Taking the three latent profiles as dependent variables, the variables with statistical significance in the univariate analysis were included in the binary logistic regression model. The results of the binary logistic regression are shown in **Table 4**.

**Table 4:** Multivariable logistic regression analysis of latent profiles of quality of life management capability in elderly frail patients with colorectal cancer.

Group comparison	Item	*OR*	*95% CI*	*P*
Profile 1 vs. Profile 2¹)	SSRS score	1.841	(1.021, 3.322)	0.041
	SUPPH scale score	1.283	(1.061, 1.553)	0.012
	HADS score	0.686	(0.524, 0.898)	0.007
	Sex (Female)	0.501	(0.258, 0.974)	0.041
Profile 1 vs. Profile 3¹)	Educational level (primary school)	0.140	(0.045, 0.435)	0.001
	Average monthly household income (<3,000 RMB)	0.976	(0.963, 0.998)	0.005
	Tumor stage (Tis stage)	1.041	(1.016, 1.075)	0.004
	SUPPH score	1.063	(1.025, 1.102)	0.001
	HADS score	0.74	(0.597, 0.917)	0.006
Profile 2 vs. Profile 3²)	Educational level (senior high school)	1.209	(1.002, 1.458)	0.047
	Average monthly household income (>6,000 RMB)	1.266	(1.012, 1.855)	0.021
	Tumor stage (Tis–Stage I)	1.210	(1.045, 1.445)	0.021
	Presence of intestinal stoma	0.481	(0.260, 0.889)	0.022
	SSRS score	1.212	(1.016, 3.526)	0.047

Profile 1: Low QOL, low emotional level group; Profile 2: Moderate QOL, low social function group; Profile 3: High QOL, high role management group.

SUPPH, Self-management efficacy scale for cancer; HADS, Hospital Anxiety and Depression Scale; SSRS, Social Support Rating Scale; AIS-CHI, Chinese Version of Acceptance Illness Scale.

¹) Reference group: Profile 1; ²) Reference group: Profile 2..

## 4 Discussion

### 4.1 Heterogeneity in quality of life management capability among elderly frail patients with colorectal cancer

Using LPA, this study identified three distinct latent profiles with significant heterogeneity in quality of life (QOL) management capability among elderly frail patients with colorectal cancer.

Low QOL, low emotional level group (20.35%): This group had the lowest overall QOL and emotional function scores, which may be associated with lower levels of social support.

Moderate QOL, low social function group (39.28%): Patients in this group scored relatively low in the social function dimension, indicating limited ability and willingness to engage in daily social interactions.

High QOL, high role management group (40.35%): This group achieved the highest scores in the role function dimension, suggesting that patients were more willing and able to fulfill their social and family roles.

Therefore, healthcare providers should implement positive psychological interventions to address negative emotions among patients in the low QOL, low emotional level group and collaborate with family caregivers to improve social support for these patients. For patients in the moderate QOL, low social function group, healthcare providers should organize peer education lectures and patient exchange meetings to enhance their willingness to engage in social interactions.

### 4.2 Higher likelihood of belonging to the low QOL, low emotional level group among specific patient populations

This study found that elderly frail patients with colorectal cancer who had low illness acceptance, low self-management efficacy, low social support, primary school education or below, average monthly household income <3,000 RMB, and of the female sex were more likely to be classified into the low QOL, low emotional level group.

Patients with low illness acceptance tend to experience negative emotions, exhibit poor adaptability, and are more prone to abandoning recommended cancer treatment regimens (14). Low educational level and insufficient social support are also key factors affecting illness acceptance in cancer patients, which is consistent with the findings of Haase et al. (15). In addition, the high cost of cancer treatment imposes both objective financial burdens and subjective financial distress on patients and their families, thereby reducing treatment adherence and impairing QOL (16). Furthermore, female patients may be more susceptible to developing negative emotional responses when confronting illness.

Thus, healthcare providers should pay special attention to female patients with low educational attainment and low income. By explaining disease-related information and treatment plans in clear, accessible language, healthcare providers can help these patients improve their mental state and actively participate in treatment.

### 4.3 Higher likelihood of belonging to the moderate QOL, low social function group among patients with intestinal stoma

This study revealed that elderly frail patients with colorectal cancer who had an intestinal stoma were more likely to be categorized into the moderate QOL, low social function group. This may be attributed to the thin, fragile, and wrinkled abdominal skin commonly observed in elderly frail patients with intestinal stomas, which increases the risk of stoma-related complications and peristomal skin issues (17). Such complications often lead to stoma aversion, trigger psychological difficulties such as social avoidance, and ultimately impair patients’ social functioning (18).

Therefore, healthcare providers should strengthen nursing guidance, provide training and one-on-one instruction to improve patients’ self-care abilities. Psychological counseling services should also be incorporated to help patients overcome negative emotions, integrate into society, and enhance their QOL.

### 4.4 Higher likelihood of belonging to the high QOL, high role management group among patients with early-stage tumor (Tis–Stage I) and no intestinal stoma

The study found that elderly frail patients with colorectal cancer who had an early tumor stage (Tis–Stage I) and no intestinal stoma were more likely to be classified into the high QOL, high role management group. As a stable indicator for predicting patients’ survival outcomes, tumor stage is a key factor influencing self-management capability (19). Patients with early-stage disease typically have a more favorable prognosis and receive more positive psychological reinforcement from their treatment experience.

Therefore, when formulating self-management strategies, healthcare providers should consider both tumor stage and the presence of an intestinal stoma, with special attention given to patients with advanced-stage cancer or stomas. Targeted guidance and support should be provided to improve these patients’ health status and QOL.

## 5 Limitations and prospects

Given that this study only collected data at a single time point and did not account for the dynamic changes of the disease, future longitudinal studies are needed to examine the trajectories of QOL management capability over time and its influencing factors. Due to the cross-sectional design, the multivariable regression results should be interpreted as exploratory associations rather than causal relationships.

## 6 Conclusion

Using LPA, this study classified the self-management behaviors of elderly frail patients with colorectal cancer into three latent profiles: low QOL, low emotional level; moderate QOL, low social function; and high QOL, high role management. These findings demonstrate significant heterogeneity in QOL management capability within this population. It is recommended that healthcare providers develop targeted nursing interventions based on the characteristics of patients in each profile.

## Data Availability

The raw data supporting the conclusions of this article will be made available by the authors, without undue reservation.
